# Application of Sulfinate Agent in Conjunction with HOCl Smear-Layer Deproteinization Improves Dentin Bonding Durability of One-step Self-etch Adhesives

**DOI:** 10.3290/j.jad.b2920099

**Published:** 2022-04-14

**Authors:** Kittisak Sanon, Antonin Tichy, Ornnicha Thanatvarakorn, Taweesak Prasansuttiporn, Kazuhide Yonekura, Keiichi Hosaka, Masayuki Otsuki, Masatoshi Nakajima

**Affiliations:** a PhD Candidate, Department of Cariology and Operative Dentistry, Tokyo Medical and Dental University, Tokyo, Japan; Department of Operative Dentistry, Faculty of Dentistry, Chulalongkorn University, Bangkok, Thailand. Principal investigator, statistical analysis, wrote the manuscript, contributed substantially to discussion.; b Assistant Professor, Institute of Dental Medicine, First Faculty of Medicine of the Charles University and General University Hospital in Prague, Prague, Czech Republic. Consulted on the idea, wrote the manuscript, contributed substantially to discussion.; c Clinical Lecturer, Faculty of Dentistry, Bangkokthonburi University, Bangkok, Thailand. Consulted on the idea, proofread the manuscript, contributed substantially to discussion.; d Associate Professor, Department of Restorative Dentistry and Periodontology, Faculty of Dentistry, Chiang Mai University, Chiang Mai, Thailand. Consulted on the idea, proofread the manuscript, contributed substantially to discussion.; e Assistant Professor, Department of Regenerative Dental Medicine, Tokushima University Graduate School of Biomedical Sciences, Tokushima, Japan. Consulted on the idea, proofread the manuscript, contributed substantially to discussion.; f Professor, Department of Regenerative Dental Medicine, Tokushima University Graduate School of Biomedical Sciences, Tokushima, Japan. Idea, hypothesis, resource management, proofread the manuscript.; g Associate Professor, Department of Cariology and Operative Dentistry, Tokyo Medical and Dental University, Tokyo, Japan. Idea, hypothesis, resource management, proofread the manuscript.; h Adjunct Lecturer, Department of Cariology and Operative Dentistry, Tokyo Medical and Dental University, Tokyo, Japan. Idea, hypothesis, experimental design, data interpretation, proofread the manuscript, contributed substantially to discussion.

**Keywords:** adhesion to dentin, all-in-one adhesive, hypochlorous acid, longevity, microtensile bond strength, resin-dentin bond strength, touch-cure activator

## Abstract

**Purpose:**

To evaluate the effect of a sulfinate agent on the bonding durability of one-step self-etch adhesives (1-SEAs) to smear-layer-covered dentin deproteinized with hypochlorous acid (HOCl).

**Materials and Methods:**

Human coronal dentin disks with a standardized smear layer were deproteinized with 100 ppm HOCl solution for 0 s (control), 15 s or 30 s. After rinsing with water for 30 s and air drying, half of the specimens were treated with a sulfinate agent (Scotchbond Universal Dual Cure Activator; SDA) prior to the application of a 1-SEA (Bond Force II [Tokuyama Dental] or Clearfil Universal Bond Quick [Kuraray Noritake]). Microtensile bond strength (µTBS) was measured after 24 h or 10,000 thermal cycles (TC). The data were analyzed by three-way ANOVA with Tukey’s post-hoc tests and t-tests at the 0.05 significance level.

**Results:**

The 24-h µTBS of both adhesives increased statistically significantly with the HOCl pretreatment for 15 s or 30 s (p < 0.05), but it was not statistically significantly affected by the application of SDA (p > 0.05). However, after TC, the groups treated with the combination of HOCl and SDA maintained their µTBS (p > 0.05), as opposed to untreated dentin and dentin treated with either HOCl or SDA, whose µTBS decreased significantly (p < 0.05).

**Conclusion:**

The application of the sulfinate agent did not statistically significantly affect the immediate bond strength of 1-SEAs, and it could not prevent a significant decrease in the bond strength to untreated dentin after thermocycling. However, the sulfinate agent significantly improved the bonding durability of 1-SEAs to HOCl smear-layer deproteinized dentin.

Self-etch adhesives (SEAs) are widely used in clinical practice, because their bonding performance is comparable to etch-and-rinse adhesives,^29^ but their application procedure reduces technique sensitivity. While two-step self-etch adhesives (2-SEAs) are considered the gold standard, one-step self-etch adhesives (1-SEAs) are popular due to their simpler, faster application procedure. However, adhesive layers formed by 1-SEAs contain hydrophilic monomers and remnants of solvents, and the consequent increased hydrophilicity has been associated with hydrolytic degradation and impaired bonding durability.^7,44^ To address this issue, 2-hydroxyethyl methacrylate (HEMA) was eliminated from some adhesives or substituted with hydrophilic amide monomers,^18,30^ and the subsequent application of a hydrophobic bonding agent improved the bonding durability of 1-SEAs as well.^2,43,46^

Bonding of SEAs is influenced by the presence of a smear layer, ie, preparation debris lacking morphological, physiological, and chemical connection with the underlying dentin. The dentin smear layer is primarily composed of disorganized collagen debris binding mineral particles, and the gelatinous collagen matrix around the mineral particles makes it difficult even for phosphoric acid to dissolve them.^35^ SEAs are less acidic and as a result, the smear layer is only partially removed,^35,38^ and the residual debris is incorporated in the adhesive layer of SEAs, forming the so-called hybridized smear layer above the authentic hybrid layer.^38^ Since the remaining organic debris within the hybridized smear layer cannot form chemical bonds or a mechanical connection with the adhesives, the hybridization of the smear layer is considered undesirable. Moreover, the smear layer acts as a barrier to monomer infiltration and interferes with the chemical interaction of adhesive monomers with the underlying intact dentin.^23,36^ Some demineralized collagen fibrils of the underlying dentin may be insufficiently sealed by the adhesive, becoming prone to hydrolytic and enzymatic degradation,^3,6^ and it was shown to compromise the long-term bond stability of 1-SEAs and 2-SEAs.^32,33^

Recently, smear-layer deproteinization with sodium hypochlorite (NaOCl) and hypochlorous acid (HOCl) has been introduced to avoid formation of the hybridized smear layer by dissolving the organic phase of the smear layer.^12,28^ The pretreatment with NaOCl and HOCl was also found to reduce the smear layer thickness, thus facilitating the infiltration of adhesive monomers into the underlying dentin and reducing nanoleakage formation.^40,41^ Moreover, the removal of the hydrated organic phase increases the inorganic-to-organic ratio and decreases the water content on the smear layer-covered dentin surface,^15,26,40,49^ enhancing the chemical interaction between adhesive monomers and hydroxyapatite of the underlying dentin.^49^ However, it was reported that the oxidizing effect of deproteinizing agents, especially NaOCl, hampers the polymerization of adhesives by premature chain termination, leading to compromised bond strength.^19,25^

An additional application of a reducing agent or antioxidant was found effective in neutralizing the oxidizing effect of deproteinizing agents, leading to the recovery of the bond strength.^24,31-33^ This was previously used in endodontics after irrigation with NaOCl, and a sulfinate-containing agent (Accel, Sun Medical; Kyoto, Japan) was commercialized as a pretreatment agent for root canal dentin, aiming at improving the adhesion of root canal sealers. Accel was also reported to improve the dentin bonding durability of a two-step self-etch adhesive (2-SEA), regardless of the application of NaOCl.^32,33^ More recently, the derivatives of sulfinic or sulfonic acid have been introduced as touch-cure activators. They serve as initiators of polymerization under the condition of insufficient photo-irradiation, eg, when bonding to root canal dentin or luting of indirect restoration.^14,47^ Sulfinates and sulfonates can also prevent the adverse reaction between acidic monomers of 1-SEAs and tertiary amines, which are co-initiators of benzoyl peroxide (BPO) in self-/dual-cure resin composites.^22,39^ Besides improving the polymerization and bonding performance of 1-SEAs under no or limited photo-irradiation, a recent study demonstrated the pretreatment of dentin with touch-cure activators can increase the degree of conversion (DC) of 1-SEAs even if they are sufficiently light cured.^11^

While these pretreatments are time consuming and diminish the user-friendliness of 1-SEAs, recent 1-SEAs labeled as universal may offer other advantages over multi-step adhesives, such as bonding in different etching modes and adhesion to various substrates without additional primers.^27^ To date, there are only a few studies on the effect of dentin pretreatment with sulfinate agent in conjunction with or without smear-layer deproteinization on the bonding of 1-SEAs to dentin.^31-33,40,41^ Therefore, this study aimed to evaluate the effect of a sulfinate agent on the bonding performance of two 1-SEAs to untreated and HOCl-deproteinized dentin after 24 h and 10,000 thermal cycles. The tested 1-SEAs differed in the content of 10-methacryloloxydecyl dihydrogen phosphate (10-MDP) and hydrophilic monomers. HOCl was selected as the deproteinizing agent, because it is easier to be washed away and leaves fewer radicals on the treated surface than NaOCl.^1^ Consequently, HOCl does not affect the polymerization of adhesives as much as NaOCl.^17,40^ Furthermore, the antimicrobial, oxidizing and deproteinizing properties of HOCl are higher compared to NaOCl, even at a much lower chlorine concentration.^10,21,45^ The null hypothesis was that neither smear-layer deproteinization with HOCl nor the application of sulfinate agent would improve the dentin bonding durability of the 1-SEAs.

## Materials and Methods

The materials used in this study (Table 1) included two 1-SEAs (Bond Force II [BF2], Tokuyama Dental; Tsukuba, Japan, and Clearfil Universal Bond Quick [UBQ], Kuraray Noritake; Tokyo, Japan), a sulfinate-containing touch-cure activator (Scotchbond Universal Dual Cure Activator [SDA], 3M Oral Care; St Paul, MN, USA) which was used as a reducing agent, and a resin composite (Clearfil AP-X; Kuraray Noritake). A 100-ppm HOCl solution was used as a deproteinizing agent. It was prepared by diluting a 500-ppm HOCl solution (Dent Zia; Tokuyama Dental) with distilled water, and its pH was adjusted to 6.8 with 1 N NaOH.

**Table 1:** Materials used in this study

Material (manufacturer)	Batch number	Composition	Application procedure
Bond Force II (Tokuyama Dental; Tsukuba, Japan)	130	Self-reinforcing phosphoric acid monomer, bis-GMA, TEG-DMA, HEMA, alcohol, water, camphorquinone, sodium fluoride	1. Apply adhesive and wait for 10 s 2. Dry with gentle air stream for 5 s 3. Light cure for 10 s
Clearfil Universal Bond Quick (Kuraray Noritake; Tokyo, Japan)	6K0215	10-MDP, bis-GMA, HEMA, hydrophilic amide monomer, colloidal silica, ethanol, dl-camphorquinone, accelerators, water, sodium fluoride	1. Apply adhesive with rubbing motion (no waiting time) 2. Dry with gentle air for 5 s 3. Light cure for 10 s
Scotchbond Universal Dual Cure Activator (3M Oral Care; St Paul, MN, USA)	3466529	Ethanol, sodium p-toluenesulfinate, methyl ethyl ketone	1. Apply activator to dentin and wait for 5 s 2. Dry with gentle air for 5 s
Clearfil AP-X (Kuraray Noritake)	AV0104	Bis-GMA, TEG-DMA, camphoquinone, photoinitiators, pigments, silanated barium glass, silanated silica	1. Apply resin composite in thickness less than 2 mm 2. Light cure for 20 s 3. Repeat 3 times

Bis-GMA: bisphenol A-glycidylmethacryalte; TEG-DMA: triethylene glycol dimethacrylate; HEMA: 2-hydroxyethyl methacrylate; 10-MDP: 10-methacryloloxydecyl dihydrogen phosphate.

### Specimen Preparation

**Fig 1:**
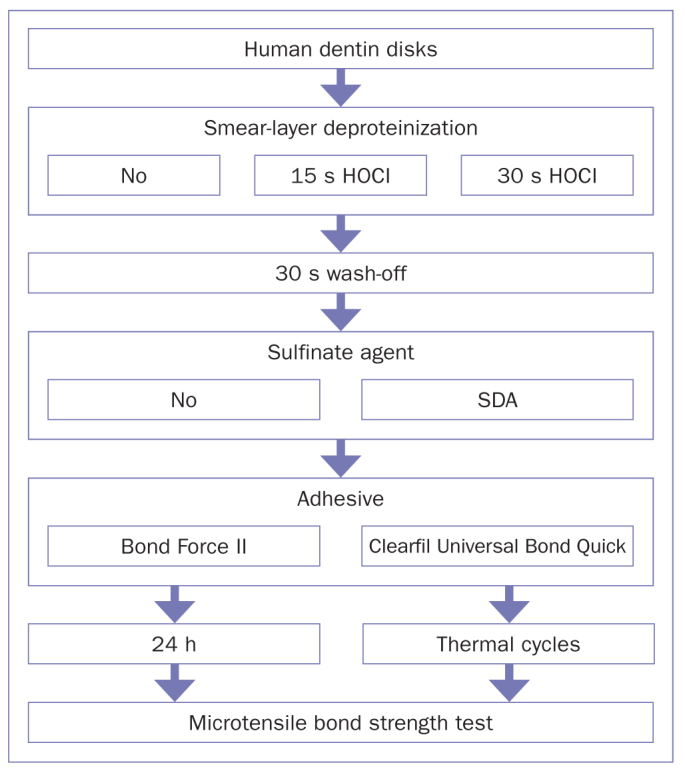
Schematic illustration of the study design. SDA: Scotchbond Universal Dual Cure Activator.

The ethics committee of Tokyo Medical and Dental University approved this study (protocol number 2013-022). One hundred twenty extracted, sound, human third molars were collected and stored in periodically changed distilled water at 4°C for no longer than six months. Their occlusal surfaces were ground flat perpendicular to the long axis of the tooth using a model trimmer under water cooling. A standardized smear layer was created on the exposed dentin surfaces using a 600-grit SiC paper under running water for 30 s. The specimens were randomly divided into 3 groups according to the application time of the 100 ppm HOCl solution: 0 s (no deproteinization, control group), 15 s, or 30 s. After the pretreatment, the dentin surfaces were rinsed with running water for 30 s and air dried for 15 s. Half of the dentin surfaces were then treated with SDA for 5 s, followed by another 5 s of air drying. Finally, the 1-SEAs (BF2 or UBQ) were applied according to the respective manufacturer’s instructions (10 teeth per group, Table 1) and light cured for 10 s (1000 mW/cm^2^, Valo, Ultradent; South Jordan, UT, USA). Three increments of Clearfil AP-X resin composite were placed on the bonded dentin surface, each of which was light cured for 20 s (1000 mW/cm^2^, Valo, Ultradent). The specimens were stored in water at 37ºC for 24 h. The study design is schematically presented in Fig 1.

### Microtensile Bond Strength (µTBS)

Each bonded specimen was sectioned parallel to the long axis of the tooth into stick-shaped specimens (bonded surface area of 1.0 ± 0.1 mm^2^) using a low-speed diamond saw with water cooling (Isomet, Buehler; Lake Bluff, IL, USA). Four sticks from the central part of each bonded specimen were used. The sticks were randomly divided into two groups of 20 sticks: 1. 24 h water storage; 2. artificial aging by 10,0000 thermal cycles (TC). Thermocycling was done in accordance with the Academy of Dental Materials guidance on μTBS testing,^4^ ie, between 5°C and 55°C, with a dwell time of 30 s in each bath and a transfer time of 5 s. After the designated aging procedure, sticks were attached to a universal testing machine (EZ-SX Test, Shimadzu; Kyoto, Japan) and subjected to the μTBS test at a crosshead speed of 1 mm/min.

In the statistical analysis, the sticks were considered statistical units (n=20). Since μTBS values were normally distributed and their variance was homogeneous, as indicated by the Kolmogorov-Smirnov test and Levene’s test, respectively, the data acquired after 24 h and TC were separately analyzed using a three-way ANOVA with Tukey’s post-hoc tests. The bonding durability was analyzed by comparing μTBS after 24 h and TC in each group using t-tests. The analyses were performed at a significance level of 0.05 using SPSS version 27.0 (IBM; Armonk, NY, USA).

### Failure Mode Analysis

After the μTBS test, both the dentin and composite sides of the fractured specimens were desiccated, sputter-coated with gold, and observed using a scanning electron microscope (SEM; JSM-IT100, JEOL; Tokyo, Japan). Failure modes were classified as follows: adhesive failure (>80% of the fracture occurred between the adhesive and dentin); cohesive failure in dentin (>80% of the fracture occurred in the dentin); cohesive failure in resin (>80% of the fracture occurred in the adhesive and/or the overlying resin composite); mixed failure (combination of adhesive and cohesive failure, each <80% of the fracture). The percentage of surface area was estimated by superimposing a 10 x 10 table on the SEM photomicrographs.^41^ The failure mode percentages were statistically analyzed using the non-parametric Pearson chi-squared test at a significance level of 0.05.

### SEM Observation of Smear-Layer-covered Dentin Surfaces

Six additional flat dentin surfaces with a standardized smear layer were prepared as described above. They were randomly divided into 3 groups according to the HOCl-application time: 0 s (control group), 15 s, or 30 s (n = 2). After pretreatment, the specimens were rinsed with water for 30 s and dehydrated in an ascending series of ethanol concentrations: 25%, 50%, and 75%, each for 20 min, 95% ethanol for 30 min, and 100% ethanol for 60 min. The specimens were then immersed in hexamethyldisilazane (HMDS) for 10 min and dried in a desiccator for 24 h at room temperature. The desiccated specimens were then fractured in half using a scalpel blade and a hammer. After gold sputter-coating, the specimens were observed using the SEM (JSM-IT100, JEOL) at a magnification of 2000X.

## Results

### µTBS

The results of the µTBS test are summarized in Table 2. Three-way ANOVA of the 24-h results revealed that initial µTBS was influenced by HOCl application time (p < 0.001) and adhesive (p < 0.001), but not by the application of SDA (p = 0.14). There was a significant interaction between HOCl-application time and adhesive (p < 0.001), the interaction between other factors were not significant (p > 0.05). After TC, µTBS was significantly influenced by all three factors (p < 0.001). The two-way interactions were highly significant as well (p < 0.001), whereas the significance of the three-way interaction was weak (p = 0.04).

**Table 2:** Means and standard deviations of microtensile bond strength (MPa)

Adhesive	Pretreatment		24 h	TC
	HOCl	Sulfinate agent		
BF2	No	No	59.9 (3.9)^A,a^	49.7 (4.6)^A,b^
		SDA	60.9 (5.8) ^A,a^	51.7 (3.8)^A,b^
	15 s	No	73.9 (4.9)^B,a^	51.9 (3.9)^A,b^
		SDA	76.2 (4.1)^B,a^	75.8 (3.2)^B,a^
	30 s	No	74.4 (3.9)^B,a^	52.0 (5.8)^A,b^
		SDA	76.2 (3.8)^B,a^	75.5 (4.1)^B,a^
UBQ	No	No	76.9 (4.5)^B,a^	68.5 (4.2)^C,b^
		SDA	76.2 (5.3)^B,a^	68.9 (3.8)^C,b^
	15 s	No	85.4 (5.9)^C,a^	66.1 (4.6)^C,b^
		SDA	85.9 (5.0)^C,a^	85.1 (4.2)^D,a^
	30 s	No	85.7 (3.4)^C,a^	69.1 (3.4)^C,b^
		SDA	86.2 (5.1)^C,a^	84.4 (4.5)^D,a^

Significant differences in each column are indicated by different superscript capital letters. Significant differences in each row are indicated by different superscript lowercase letters. BF: Bond Force II (Tokuyama Dental); UBQ: Clearfil Universal Bond Quick (Kuraray Noritake); SDA: Scotchbond Universal Dual Cure Activato (3M Oral Care); TC: 10,000 thermal cycles.

HOCl pretreatment and SDA application had similar effects on the µTBS of both adhesives. After 24 h, pretreatment with HOCl for 15 s or 30 s increased μTBS significantly (p < 0.05), while the application of SDA did not significantly affect the initial µTBS, irrespective of pretreatment of HOCl (p > 0.05). However, the t-test revealed that µTBS in groups pretreated with the combination of HOCl and SDA did not change significantly after TC (p > 0.05), as opposed to untreated dentin or dentin treated with either HOCl or SDA, where a significant decrease in µTBS was found (p < 0.05).

### Failure Mode Analysis

**Fig 2:**
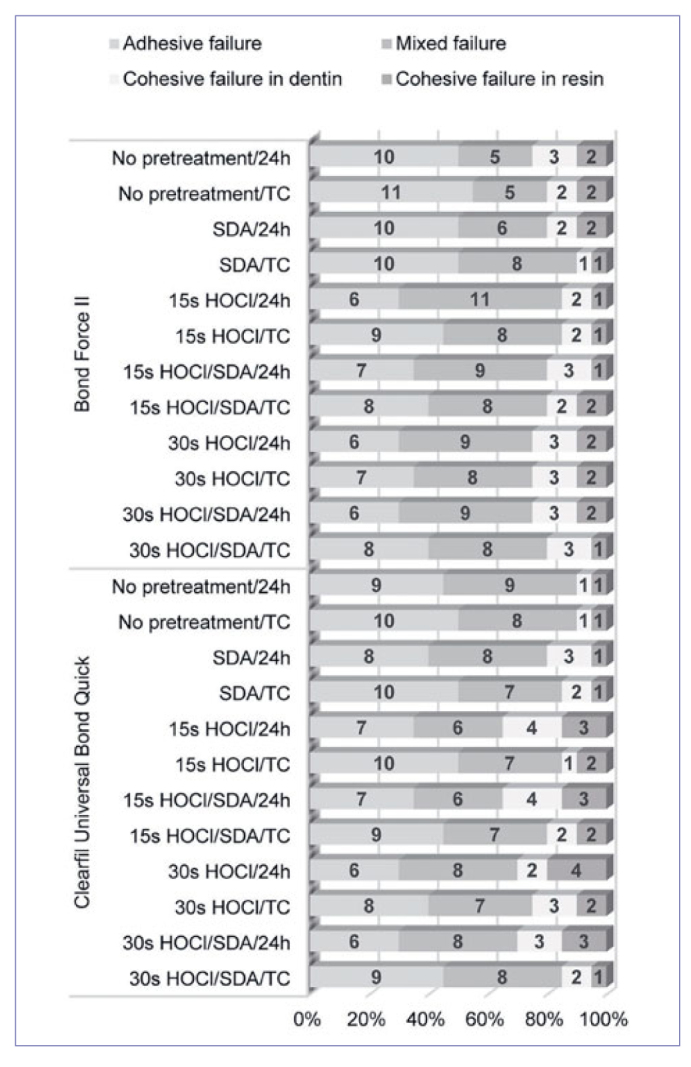
The distribution of failure modes in each group. In all groups, adhesive and mixed failures prevailed, and the distribution of failure modes was statistically similar (p = 1.00). The numbers in bars indicate the number of specimens with the respective failure mode. SDA: Scotchbond Universal Dual Cure Activator; 24 h: after storage for 24 h; TC: after 10,000 thermal cycles.

The distribution of failure modes is summarized in Fig 2. In all tested groups, the majority of failures was adhesive or mixed. There were no significant differences in failure mode distribution between the groups (p = 1.00).

### SEM Observations

**Fig 3:**
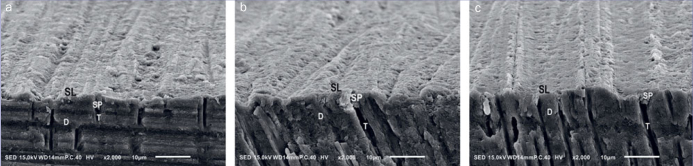
SEM images of the smear-layer-covered dentin surface. (a) No pretreatment (control); (b) smear-layer deproteinization with 100 ppm HOCl for 15 s; (c) smear-layer deproteinization with 100 ppm HOCl for 30 s. There were no obvious differences in smear-layer surface morphology between the pretreatments. D: dentin; SL: smear layer; SP: smear plug; T: dentinal tubule.

Figure 3 shows representative SEM images of the smear-layer-covered dentin surfaces without smear-layer deproteinization (Fig 3a) and treatment with HOCl for 15 s (Fig 3b) or 30 s (Fig 3c). No alteration in the surface morphology of the smear-layer-covered dentin surface after pretreatment with HOCl was observed.

## Discussion

The results revealed that the application of the sulfinate agent did not significantly affect the initial µTBS, whereas smear-layer deproteinization using HOCl significantly improved it. However, neither of the pretreatments was able to prevent a significant decrease in µTBS after TC when applied separately. The dentin bonding durability of 1-SEAs was only improved if the sulfinate agent was applied after smear-layer deproteinization. Therefore, the null hypothesis was partially rejected.

Smear-layer deproteinization with NaOCl and HOCl solutions was tested in numerous studies, but its benefits for bonding of self-etch adhesives, such as avoiding the formation of the hybridized smear layer,^40^ were often outweighed by the adverse effect of the oxidizing agent on the polymerization of adhesives.^19,25^ This problem was resolved by the subsequent application of a reducing agent or antioxidants, which can recover the compromised bond strength.^31-33^ Among the oxidizing agents, HOCl was found to be more effective in smear-layer deproteinization than NaOCl, because of higher deproteinizing ability even at lower chloride concentration and easier washing off of treated surfaces.^10,21,45^ The present results showed that the initial bond strength of both tested 1-SEAs improved significantly after pretreatment with HOCl for 15 s or 30 s, which suggested that avoiding the hybridization of the smear layer is beneficial for the initial bonding of 1-SEAs. As the increase in µTBS was observed regardless of the subsequent application of the sulfinate agent, we speculate that the wash-out time of 30 s was sufficient to reduce the adverse effect of oxidizing agents on the polymerization of 1-SEAs.

Sulfinate agents are primarily used to initiate polymerization under insufficient photo-irradiation and to resolve the incompatibility between acidic monomers of self-etch adhesives and self-/dual-cure composites.^5,13,16,20^ A recent study demonstrated that the application of sulfinate agents to dentin prior to bonding with corresponding 1-SEAs could increase their DC, even when sufficient light energy was delivered.^11^ However, when used in self-etch mode, the application of sulfinate agents did not significantly affect the µTBS of adhesives to dentin,^11,32,33^ which was confirmed by the present study, as the pretreatment with SDA alone had no significant effect on the µTBS of UBQ or BF2. Considering these results, smear-layer deproteinization with HOCl followed by a 30-s wash-out time seems to be more effective than pretreatment with sulfinate agents.

No morphological alteration of the smear-layer-covered dentin was observed using SEM, but the observation of adhesive-dentin interfaces using transmission electron microscopy in previous studies revealed that smear-layer deproteinization using HOCl removed the hybridized smear layer.^40,41^ However, it was mentioned that the use of HOCl without the application of a sulfinate agent (Accel) resulted in greater nanoleakage within the hybrid layer of 1-SEAs than if Accel was subsequently applied.^41^ In this study, the µTBS of both 1-SEAs decreased significantly after TC if the sulfinate agent (SDA) was not subsequently applied on smear-layer-deproteinized dentin, whereas groups receiving SDA maintained their µTBS. As nanoleakage pathways allow water to penetrate into the hybrid layer and hence increase its susceptibility to hydrolytic degradation,^9,37^ we presumed that the decrease in nanoleakage formation contributed to the stable µTBS in groups where HOCl pretreatment was combined with the sulfinate agent. Additionally, SDA can neutralize the HOCl-oxidized dentin surface due to its reducing properties,^24,31-33^ thus increasing the DC of 1-SEAs,^11^ which was recently reported to be positively correlated with their µTBS to dentin, especially after TC.^42^ Their higher DC presumably contributed to the durability of 1-SEAs by increasing their resistance to water sorption,^34^ in conjunction with avoiding the formation of the hybridized smear layer by deproteinization with HOCl.

The pretreatments had the same effect on both tested adhesives, but the µTBS of UBQ was significantly higher than that of BF2 in all groups. This was perhaps related to the presence of 10-MDP and a hydrophilic amide monomer in UBQ. 10-MDP is used in most contemporary 1-SEAs, because it forms very stable calcium salts with hydroxyapatite and assembles into nanolayers at the interface.^8,48^ Therefore, 10-MDP contributed to the observed difference in µTBS, as BF2 is based on a self-reinforcing phosphoric acid monomer. As for the hydrophilic amide monomer, it partially substitutes HEMA in UBQ, and it was previously shown to increase the µTBS of UBQ and decrease its water sorption compared to an experimental version containing only HEMA.^18^

## Conclusions

The application of the sulfinate agent did not significantly affect the immediate bonding of 1-SEAs to untreated and HOCl smear-layer deproteinized dentin. The sulfinate agent did not prevent a significant decrease in the bond strength to untreated dentin after thermocycling, but it significantly improved the bonding durability of 1-SEAs to HOCl-smear-layer deproteinized dentin.

## Supplement

**Table:** **Supplement 1** Result of the three-way ANOVA showing the effect of independent variables on the µTBS after 24-h storage

Kolmogorov-Smirnov normality test					
Adhesive	HOCl	Sulfinate agent	Statistic	df	p-value
BF2	No	No	0.194	20	0.05
		SDA	0.175	20	0.11
	15 s	No	0.169	20	0.14
		SDA	0.145	20	0.20
	30 s	No	0.107	20	0.20
		SDA	0.147	20	0.20
UBQ	No	No	0.094	20	0.20
		SDA	0.141	20	0.20
	15 s	No	0.109	20	0.20
		SDA	0.090	20	0.20
	30 s	No	0.133	20	0.20
		SDA	0.164	20	0.17

BF2: Bond Force II; SDA: Scotchbond Universal Dual Cure Activator; UBQ: Clearfil Universal Bond Quick.

**Table:** 

Levene’s test of equality of variance				
	Levene statistic	df1	df2	p-value
Based on mean	1.306	11	228	0.22
Based on median	1.070	11	228	0.39
Based on median and with adjusted df	1.070	11	194.288	0.39
Based on trimmed mean	1.270	11	228	0.24

**Table:** 

Test of between-subject effects					
Source	Sum of Squares	df	Mean Square	F	p-value
Main effects					
A: HOCl application time	7664.7	2	3832.4	172.9	< 0.001
B: SDA	48.9	1	48.9	2.2	0.14
C: Adhesive	9326.1	1	9326.1	420.9	< 0.001
Interaction					
AB	16.4	2	8.2	0.3	0.691
AC	407.6	2	203.8	9.1	< 0.001
BC	43.3	1	43.3	1.9	0.16
ABC	0.6	2	0.3	0.0	0.98

HOCl-application time (5 s, 15 s, or 30 s); SDA (with or without); adhesive (Bond Force II or Clearfil Universal Bond Quick). SDA; Scotchbond Universal Dual Cure Activator.

**Table:** **Supplement 2** Result of the three-way ANOVA showing the effect of dependent variables on the µTBS after thermal cycles

Kolmogorov-Smirnov normality test					
Adhesive	HOCl	Sulfinate agent	Statistic	df	p-value
BF2	No	No	0.125	20	0.20
		SDA	0.101	20	0.20
	15 s	No	0.185	20	0.07
		SDA	0.115	20	0.20
	30 s	No	0.133	20	0.20
		SDA	0.166	20	0.15
UBQ	No	No	0.121	20	0.20
		SDA	0.100	20	0.20
	15 s	No	0.105	20	0.20
		SDA	0.114	20	0.20
	30 s	No	0.135	20	0.20
		SDA	0.151	20	0.20

BF2: Bond Force II; SDA: Scotchbond Universal Dual Cure Activator; UBQ: Clearfil Universal Bond Quick.

**Table:** 

Levene’s test of equality of variance				
	Levene statistic	df1	df2	p-value
Based on mean	1.165	11	228	0.31
Based on median	0.955	11	228	0.49
Based on median and with adjusted df	0.955	11	195.606	0.49
Based on trimmed mean	1.141	11	228	0.33

**Table:** 

Test of between-subject effects					
Source	Sum of Squares	df	Mean Square	F	p-value
Main effects					
A: HOCl-application time	5645.5	2	2822.7	158.2	< 0.001
B: SDA	11771.3	1	11771.3	659.7	< 0.001
C: Adhesive	12159.6	1	12159.6	681.5	< 0.001
Interaction					
AB	4995.311	2	2497.656	139.990	< 0.001
AC	439.350	2	219.675	12.312	< 0.001
BC	364.824	1	364.824	20.448	< 0.001
ABC	110.624	2	55.312	3.100	0.04

HOCl-application time (5 s, 15 s, or 30 s); SDA (with or without); adhesive (Bond Force II or Clearfil Universal Bond Quick). Abbreviation: SDA, Scotchbond Universal Dual Cure Activator.

## Clinical relevance:

The combination of smear-layer deproteinization with HOCl and the application of a sulfinate agent could improve the dentin bonding durability of 1-SEAs.
